# Is Stress in Contact Centers Inevitable?

**DOI:** 10.3390/ijerph18062999

**Published:** 2021-03-15

**Authors:** Diogo Gonçalves-Candeias, Maria José Chambel, Vânia Sofia Carvalho

**Affiliations:** CICPSI, Faculdade de Psicologia, Universidade de Lisboa, 1649-013 Lisboa, Portugal; DiogoMCandeias@hotmail.com (D.G.-C.); vscarvalho@psicologia.ulisboa.pt (V.S.C.)

**Keywords:** contact centers, stress, human resources practices, work characteristics, social support, work–personal life relationship

## Abstract

It is broadly acknowledged that contact center employees are subject to high levels of stress. In this profession, there is a distinction between back-office and front-office employees. In addition, employees may perform duties in various companies with different characteristics (i.e., human resources practices, job characteristics, social support, work–personal life relationship, among others). Thus, this study focuses on the analysis of the contact centers’ (CC) psychosocial work environment and employees’ levels of stress and well-being, seeking to understand whether they change due to the specific nature of the duties they perform and the characteristics of the company. This study involved 1440 participants from 15 companies. The results indicate that front-office and back-office duties influence the perception of some job characteristics and their environment and, consequently, the stress and well-being of these employees. Furthermore, the exhaustion and general well-being of employees are seemingly independent of the duties performed and common to all companies. However, the job characteristics, psychosocial environment and employees’ levels of cynicism, work engagement and general stress were found to change according to the company in which they worked, thus highlighting the need for action in the psychosocial environment of these work duties.

## 1. Introduction

Political, social, technological, and economic changes have brought new paradigms and challenges to the organizational world, and it is crucial that organizations adapt in order to remain competitive in the market. CC have emerged in this context and may be characterized as an industry with a high rate of growth, at both international and national levels [1]. CC have become the main form of contact and interaction with customers [2,3], whereby communication is made through a number of channels (i.e., emails, telephone calls), reducing costs for organizations and improving customer service [4]. CC therefore offer various services, ranging from problem solving (and dealing with complaints) to providing additional information [5] and fostering customer loyalty [6], as there is closer proximity between the organization and its customers [7] in so far as there are no geographical barriers and round the clock service is offered [8].

According to the Portuguese Association of Contact Centers (APCC), there are over 80,000 employees in Portugal. However, although CC contribute to a personalized and higher quality customer service, they are associated with high levels of employee turnover, absenteeism, stress and burnout [9]. These consequences are the result of monotonous and repetitive duties, comparable to a modern form of Taylorism [10], where employees have little autonomy over their work and tasks [11] which are neither complex nor challenging [12]. This simplification of work results from a structural division where, in most cases, an employee only performs front-office (answering calls) or back-office (administrative tasks) duties. The lack of rewards is compounded by low pay and a high workload which can have a negative impact on employees’ stress and well-being [13]. It should also be noted that the scripts used by these employees, with detailed instructions that structure and organize their intervention [14], enable a high level of control by the organization.

Nevertheless, some research has shown that Human Resources practices can be developed [15,16,17] which, combined with the establishment of positive relationships with the supervisor (leaders) and co-workers (peers) [18] can mitigate these negative effects on employees’ stress and well-being.

In this study, the aim is to ascertain the extent to which the different back-office and front-office duties differ both in terms of the psychosocial work environment, and the levels of stress and well-being experienced by the employees. Considering the nature of the work of front-office and back-office employees, job characteristics, social support, HR practices, work–life conflict, workplace attitudes and well-being and general well-being were compared in both duties. Additionally, this study seeks to ascertain whether these characteristics are identical across all the companies, or whether there may be differences in the tasks performed or in their management. To this end, the employees of a total sample of 15 CC companies were studied, thus making it possible to estimate the proportion of the psychosocial work environment conditions, stress and well-being attributable to the characteristics of the organization. Hence, a further aim is to clarify whether the characteristics inherent to the work of CC are inevitable or whether they depend on the duties performed (i.e., back-office and front-office) or organizational context characteristics. Since there is no theoretical development according to which it might be possible to establish potentially significant differences, this study is of an exploratory nature, seeking solely to ascertain whether there is consistency between the job, context and well-being characteristics of back and front-office employees in fifteen distinct companies.

Consequently, this study contributes to the construction of a healthier working environment, in an area characterized by constant growth.

## 2. Theoretical Framework

### 2.1. Job Characteristics

According to the JD-C (Job Demands–Control) model, the demands and control (i.e., autonomy) job characteristics can explain the stress and well-being of employees, and situations of stress occur when the job is characterized by high demands and low control [19,20,21].

Previous studies have shown that CC are associated with high demands related to the way employees are constantly monitored and evaluated [10]. The evaluation of employees is mostly based on quantitative criteria, which consider several factors such as the number of calls, their duration and also the number of calls on hold [12]. Therefore, this excessive workload, resulting from HR systems’ pressure to meet the pre-established goals of the organization and the constant monitoring [22] make CC work highly demanding.

As for autonomy, CC employees are generally considered to have low control and to be dependent on the planning and organization of the tasks they perform [8,11,23]: In addition to the fact that employees do not control when or with whom they speak, they are obliged to follow scripts that organize and structure their intervention [14].

Thus, and with recourse to the job characteristics model of Karasek [20,21], CC are predicted to be environments characterized by high demands and low control (i.e., autonomy) [2,3,10].

### 2.2. Social Support

Social support has been recognized as a way of mitigating stress and reducing the negative effects of high demands and low control situations [14,24,25,26] and the JD-C(S) (Job Demands–Control–Support) model identifies social support as a third dimension that can influence stress at work [20,21].

Social support occurs as a result of the fact that social relationships and interactions at work act as resources to combat job demands [27], as employees receive the information they require and develop different coping strategies that can be used in their daily lives [18]. Since employees with access to more resources are better able to effectively respond to any demands that may arise [2,28], the CC that promote social support may be predicted to promote employees’ resources to combat the demands of the task and therefore promote their experience of well-being and reduce their levels of stress.

### 2.3. HR Practices

In the same vein, the HR practices adopted by the organization can also contribute to the well-being of employees [16]. Employees’ perception of job characteristics (i.e., demands, control) are influenced by the Human Resource (HR) practices in place [1] which may lead to increased or decreased levels of stress [22]. Several Human Resource Management (HRM) models do not focus on employee performance alone [29,30], such as the HIM (High Involvement Management) Model, which highlights the importance of empowering employees through power, information, knowledge and rewards, while equally ensuring their performance and well-being [31].

The challenge of HRM in relation to CC lies in establishing a balance between HR control practices, geared towards standardizing the work, and HR practices compatible with the HIM model that seek to reduce employees’ stress [32,33]. Therefore, it is important to study a broad range of HR practices and analyze their impact on employees’ stress and well-being [34,35]. To this end, the recruitment and selection process, the welcoming and integration process, training opportunities, rewards and the performance evaluation process were deemed HR measures that can directly influence CC employees’ levels of stress.

#### 2.3.1. Recruitment and Selection

One way to contribute to the well-being of employees is by recruiting people with a suitable profile for the job and the organization [36]. Thus, it is important to invest in the recruitment and selection process, since it can influence the quality of the work, the interpersonal relationships of employees and also the services provided by the organization [37].

In the context of CC, and since these employees perform unchallenging, monotonous and repetitive tasks, the recruitment focus should not be on seeking highly skilled employees, but rather on identifying individuals with soft skills, thus giving priority to behavioral/social skills over technical skills [38]. On the other hand, Chapman and Webster [39] also highlight the fundamental role of recruiters in this context, since they can positively influence employees’ perception of the job characteristics. In addition to the responsibility of recruiting people with a suitable profile for the job and the organization (facilitating their integration), recruitment also contributes to employees’ adjustment of expectations and behaviors by clarifying their role in the organization [40].

#### 2.3.2. Welcoming and Integration Process

The welcoming and integration process is important, as it reduces the levels of stress of the new employees, thus providing them with a positive experience at the beginning of their new job [41] and contributing to their well-being [42]. This process fosters the construction and development of the employee–organization relationship, facilitating the sharing of information, internal communication, and team cohesion [43], while also accelerating the new employee’s adaptation, since it stimulates the acquisition of knowledge regarding the culture, values and goals of the organization [44].

#### 2.3.3. Training

Training promotes personal development [45] which contributes to the development of employees’ personal resources (i.e., self-control) [46]. Thus, through a set of duly planned learning experiences, individuals acquire new knowledge and technical skills that can facilitate the execution of their tasks, which in turn reduces job demands [47].

In fact, training can be a strategy used by HRM to alleviate stress, as the sharing of knowledge and strategies are tools which better prepare employees to respond to job demands [48].

#### 2.3.4. Compensation and Rewards

Several CC have adopted a variable salary component for all the employees who meet the pre-established objectives and goals. Batt [49] has identified this incentive compensation as an HR practice that is compatible with the HIM model, which equally values employee performance and well-being [31]. This HR practice aligns the interests of the organization with the interests of all its employees [50], contributing to job satisfaction and the well-being of employees [51] who feel that their effort is being rewarded [52].

#### 2.3.5. Performance Assessment

Performance assessment consists of the continuous monitoring process of employees’ behavior and performance, which enables an assessment of how efficiently they perform their duties [53]. CC use high levels of monitoring with quantitative and qualitative criteria by which employees are assessed [54]: These criteria include not only customer satisfaction, but also the number of calls made, their duration, and the number of calls on hold [3,4,12]. This practice ensures standardization of the job but can have a negative impact on employees’ well-being [9]. In fact, according to Deery, Iverson and Walsh [14], high monitoring levels are for several reasons associated with increased levels of stress among employees. Firstly, stress may result from high demands which may lead to a role conflict [55], as employees are expected to establish a positive relationship with customers. However, on the other hand, they are also obliged to meet quantitative criteria (i.e., quantity and speed of calls), leading to an intensified workload [8]. Moreover, there may be additional pressure to meet the pre-established goals, since in most cases CC employees work according to an incentive compensation system [56]. Secondly, the high degree of monitoring reduces employees’ autonomy, as they are obliged to follow scripts that structure and organize their interaction [57]. Finally, besides a heavy workload and limited autonomy, the constant monitoring to which they are subject also implies high emotional regulation on the part of these employees, as their performance is also assessed through customer satisfaction [15]. Employees therefore use the few resources they have to combat the additional stress they experience as a result of being observed, instead of focusing on providing a quality service [58].

However, Grant and Higgins [59] maintain that performance assessment can have a positive impact on the well-being of employees. According to these authors, monitoring can be a means of identifying training needs, thus promoting the development of employees’ new skills and knowledge, which, as previously mentioned, is associated with the reduction of stress levels [47].

In this regard, the effects of monitoring and its impact on employees’ well-being depend on how the performance assessment data is used.

### 2.4. Work–Life Conflict

According to the role conflict theory [60], an individual’s resources are finite and decrease according to the roles they play. Thus, and based on the resource scarcity (i.e., time, energy) hypothesis, role conflict arises when the demands of each domain are incompatible, and the individual is obliged to choose where to apply these resources [61]. As resources are finite, when individuals participate in one domain (i.e., work), this implies an investment of their resources (i.e., time and energy), and consequently their participation in other domains is compromised [62,63,64].

The job characteristics of CC are associated with high levels of *stress*, impairing employees’ participation in other fields.

### 2.5. Attitudes and Well-Being at Work

#### 2.5.1. Organizational Commitment

Organizational commitment is a psychological state which defines the employee’s level of identification with the organization and its objectives [65] As an attitude, organizational commitment reflects the bond which links employees to the organization for which they work [66]. According to Meyer and Allen [67], this bond may be represented in different ways (i.e., affective, normative and continuity commitment), which condition the behavior of employees. Affective commitment is negatively associated with stress [68], as employees develop a positive emotional relationship with the organization and regard its goals as being compatible with their own [69], leading to a reduction in the ambiguity of their role [70] which enhances their well-being [71].

It has been acknowledged that CC are associated with high levels of stress and therefore affective commitment may be used as a resource [72] to combat stress [73]. The studies of Schmidt [74] conclude that, due to the job characteristics of CC, employees with an emotional connection to the organization display lower burnout levels than their co-workers.

#### 2.5.2. Work Engagement

Work Engagement is a stable and persistent psychological state which reflects the well-being and motivation of employees at work [75]. According to Schaufeli and Bakker [76], it is through work engagement that employees’ energy levels are expressed, reflected in their effort and persistence in the face of difficulties (vigor), their enthusiasm, pride and inspiration (dedication) and also the intrinsic pleasure and concentration associated with the performance of their duties (absorption). Thus, it may be concluded that a workforce with high engagement may constitute a competitive advantage [77], since this variable is positively associated with job satisfaction [78], general well-being [77] and is, consequently, negatively related to stress [76].

However, engagement depends on the resources (social, physical and organizational characteristics) obtained by individuals and used in the work context [79]. Therefore, the organization should provide the resources required by all its employees in order to promote their intrinsic satisfaction and enhance their well-being.

#### 2.5.3. Burnout

According to Maslach and Leiter [80], burnout is a means of identifying stress in the workplace, reflected in the employee who has not been able to adapt to the duties/organization. This may be operationalized as a prolonged response to emotional and interpersonal stressors at work and may be analyzed through two core dimensions: Exhaustion and cynicism [75]. Exhaustion refers to feelings of extreme fatigue, emotional overload, and a lack of energy and emotional resources to perform one’s work. Cynicism consists of adopting negative, cold, and distant attitudes towards work [80].

Maslach, and Leiter [80] have identified a number of burnout risk factors such as excessive workload, lack of control, and low pay. Thus, low control, a high workload and low pay contribute to the onset of burnout in CCs [81]. Moreover, burnout is negatively associated with employee satisfaction and well-being, and positively related to stress [81]. Thus, one of the challenges faced by organizations is that of adopting measures that contribute to the reduction of burnout.

### 2.6. General Well-Being

According to Johnson, Cooper and Cartwright [82], there is a correlation between job satisfaction and the physical and psychological well-being of employees. Thus, it is important to analyze dimensions such as job characteristics, social support and the HR practices adopted by the organization in the context of CC, as these variables can explain and predict the satisfaction and general well-being of employees [11].

Considering job design and the job characteristics model [23], it may be said that work in CC is monotonous and demanding and employees have a low level of autonomy. Therefore, low job control, high job demands, and the limited diversity of tasks have a negative impact on employees’ satisfaction and are also associated with high levels of stress [12]. Although there is little flexibility in terms of monitoring and job design in CC, several studies have pointed to a solution being found in the HR practices adopted by the organization [8] and the promotion of social support, as both these features can mitigate the effects of stressors [22]. Such is also the case with organizations that implement measures to foster a work–life balance, as they increase employee satisfaction and, consequently, contribute to their general well-being [83].

## 3. Method

### 3.1. Procedure and Sample

The data collection for this study was carried out as part of a research project conducted within the scope of a partnership with the Portuguese Association of Contact Centers (APCC), with the purpose of identifying and diagnosing psychosocial risks at work in the context of CC. To such end, associated companies were contacted by APCC management to participate in the study. The employees of the CC companies who agreed to participate were notified by HR of the objectives of the study and were invited to take part in the study. Through the *SurveyMonkey* platform, a link was generated which directed participants to an online survey. Finally, the employees were informed that their participation was voluntary, confidential and anonymous.

A convenience sample was obtained, corresponding to a total of 2232 employees from 15 different CC companies, with a response rate of over 70% (ranging from 71% to 81% among the companies, corresponding to 32–432 respondents per company).

However, due to a lack of responses to some of the assessed scales, only 1440 participants were considered for the study. The characteristics of the sample are presented in Table 1, in which the characteristics of the whole sample of front-office and back-office employees are presented.

### 3.2. Measures

*Job Characteristics*. Job demands and control (i.e., autonomy) were measured by means of the Job Content Questionnaire (*JCQ*) [84], as the Portuguese version had already been used in previous studies [85]. Therefore, using a Likert scale of 1 (*I totally disagree*) to 5 (*I totally agree*), the participants responded to a questionnaire composed of 7 items that analyzed job demands (e.g., *I have too much work to do*) and 4 items referring to the level of autonomy they had at work (e.g., *I have control over what happens in my work*). Thus, high scores in these two scales indicate high demands and high autonomy, respectively. The two scales have a good rate of internal consistency, as Cronbach’s α was always above 0.7 [86] (0.88 and 0.88 for demands and 0.84 and 0.85 for autonomy, for front-office and back-office employees, respectively). The JCQ was also used to measure social support [86] through 5 items that analyzed supervisor support (e.g., *My supervisor is concerned about the well-being of his/her employees*) and 6 items regarding peer support (e.g., *The people I work with help in the accomplishment of tasks*). The participants assessed the extent to which they agreed with each statement using a Likert scale of 1 (*I totally disagree*) to 7 (*I totally agree)*. High scores correspond to a high level of supervisor support and peer support. Internal consistency rates were 0.88 and 0.89 for supervisor support and 0.86 and 0.87 for peer support, for front-office and back-office employees, respectively.

*Human resources practices*. Human resources practices were analyzed with recourse to an adaptation of the scale used by Chambel, Castanheira, and Sobral [87], based on the scales of Lepak and Snell [88], Slattery, Selvarajan, and Anderson [89], Takeuchi, Lepak and Wang [90] and Chambel and Castanheira (2012) [1]. The questionnaire consisted of a total of 22 items which analyzed the various human resources practices adopted by the organization to which the participants responded using a Likert scale of 1 (*I totally disagree*) to 7 (*I totally agree)*. Recruitment was measured by 4 items (e.g., *When I was recruited by this company my specific knowledge was analyzed*), presenting a Cronbach’s α of 0.80 and 0.83 for front-office and back-office employees, respectively. The welcoming and integration process consisted of 4 items (e.g., *When I started working in this company I had initial support from my supervisor*) and Cronbach’s α was 0.83 for front-office and 0.81 for back-office employees. Training was analyzed by means of 5 items (e.g., *With the training/experience I have received I can easily change roles within this company*), with a Cronbach’s α of 0.91 for front-office and 0.90 for back-office employees. Performance assessment was measured by 4 items (e.g., *The performance assessment criteria are clear in this company*), with a Cronbach’s α of 0.88 for front-office employees and 0.91 for back-office employees. Finally, compensation was analyzed by means of 5 items (e.g., *In this company, the criteria for assigning the variable component of the salary are clear*), with a Cronbach’s α of 0.88 and 0.90 for front-office and back-office employees, respectively.

High scores in these dimensions indicate that employees had a more positive perception of the HR practices in place.

*Work–life conflict***.** The work–life conflict was measured through the Portuguese version of the scale of Keeney, Boyd and Sinha [91], used by Chambel, Carvalho, and Cesário [92]. It considers 8 work-related domains: Health, family, home management, friendship, education, love relationships, leisure, and community involvement. However, the latter domain was not considered for this study since people in Portugal do not have a high and systematic involvement in community activities [92]. The interference of work in one’s personal life may occur in two distinct dimensions, namely time (e.g., *Work takes the time* that *I would like to spend with my family away from me*) and stress (e.g., *Due to all the pressures of work, I am sometimes too stressed to engage in family activities*). Each was measured by 7 items and participants had to assess the extent to which they agreed with each statement using a *Likert* scale of 1 (*I totally disagree*) to 5 (*I totally agree)*. Cronbach’s α for time was 0.93 for front-office and back-office employees, and for stress 0.94 and 0.95 for front-office and back-office employees, respectively.

*Affective organizational commitment*. Affective organizational commitment was measured through the Portuguese version of Meyer, Allen and Smith’s [68] scale used in the study of Chambel and Castanheira [15]. The scale is composed of 6 items (e.g., *This company has a high personal meaning to me*) that were answered using a *Likert* scale of 1 (*I totally disagree*) to 7 (*I totally agree)*. This scale also presented good internal consistency, since Cronbach’s α was 0.88 for front-office employees and 0.90 for back-office employees.

*Well-being at work.* Well-being at work was measured by work engagement and burnout. Work engagement was analyzed using the Portuguese version of the Schaufeli, Bakker and Salanova [93] scale, used previously by Chambel et al. [87]. This version consisted of 3 items to measure vigor (e.g., *In my work I feel full of energy*); 3 items for dedication (e.g., I *am enthusiastic about my work*) and 3 items for absorption (e.g., *I am immersed in my work*). Participants’ responses were measured using a *Likert* scale of 1 (*Never*) to 7 (*Everyday*) and high scores indicate high levels of work engagement. Considering the front-office and back-office employees, Cronbach’s α were 0.94 and 0.95 respectively. Burnout was measured by means of the Portuguese version of the Maslach, Jackson and Leiter [94] scale, used previously by Chambel and Castanheira [15]. This scale is composed of 5 items that analyze exhaustion at work (e.g., *I feel exhausted by my work*) and 5 items related to cynicism (e.g., *I have lost enthusiasm for my work*), both measured on a Likert scale ranging from 1 (*Never*) to 7 (*Everyday*). As with engagement, high scores indicate high burnout levels. For exhaustion, Cronbach’s α was 0.92 for both front-office and back-office employees, while for cynicism, Cronbach’s α was 0.85 and 0.84, respectively.

*General well-being*. General well-being was measured through an adapted version of the *General Health Questionnaire* (GHQ-12 [95]). This questionnaire is composed of 12 items (e.g., *Have you been feeling sad and depressed?*) and the participants responded using a Likert scale of 1 (*Not at all*) to 4 (*Much more than usual*). The scale was subdivided into two dimensions in order to analyze employees’ stress and well-being, not in a professional context, but on a general level (i.e., in a free context). Regarding internal consistency, Cronbach’s α showed no differences between front-office and back-office employees, standing at 0.84 in the stress sub-scale and 0.87 in the well-being sub-scale.

## 4. Data Analysis

The data analysis was performed through the IBM Statistical Package for the Social Sciences (SPSS 25.0, IBM, New York, NY, USA) program.

In order to characterize the sample, a descriptive analysis of variables such as gender, age, marital status, qualifications, work shift and tenure of the respondents, was conducted for the whole sample and for both front-office and back-office duties. A descriptive analysis of the instruments used was then carried out, which made it possible to calculate the main measures of central tendency and dispersion of each of the studied variables. The Student *t*-test was performed to verify whether the means of the two groups, both front-office and back-office, were statistically different. The *Cronbach’s* alpha of each scale was also calculated to analyze the internal consistency.

Finally, the intra-class correlation coefficient (ICC) was calculated to evaluate the amount of variation in the responses at the individual level for each scale that can be explained by the variability among the 15 CC companies. The intraclass correlation (ICC) was calculated to assess the amount of variance in individual-level responses for each variable that can be explained by variability among the fifteen organizations:

ICC = (msb − msw)/(msb + ((ng − 1) msw))(1)

where msb is the between-group mean square, msw is the within- group mean square, and ng is the group size, [96]. The higher the ICC value, the higher the proportion of total variance in a subscale is explained by organizational membership. When evaluating the ICC, values exceeding 0.05 are considered relevant for aggregation of individual-level data to a higher organizational level, and 0.20 is considered to be a high level. Thus, it is possible to identify which of the organization’s characteristics influence the psychosocial work environment and the stress and well-being of its employees.

## 5. Results

Table 2 shows the mean (M) and standard-deviation (SD) of the variables in the sample under study and by means of the Student *t*-test, the comparison of means between the back-office and front-office groups may be observed. On the basis of this comparison, it was possible to verify that the employees of these two groups have a similar perception in several of the factors considered, showing that the latter are independent of the duties. Thus, it was possible to observe that employees in both groups have a moderately high perception of job demands and feel that there is moderate supervisor and peer support. As far as HR practices are concerned, employees have a slightly positive perception of the integration and recruitment processes, as well as the training and assessment carried out by the organization. Employees show a weak affective commitment to the organization, relatively low work engagement, relatively high cynicism in the exercise of their professional activity and weak general well-being. However, it was possible to observe some significant differences between the two groups. Front-office employees show lower values in the perception of autonomy, higher values in compensation, work–life conflict (stress dimension) and exhaustion, but lower for general stress. Thus, and although the conditions are similar, the results appear to indicate that front-office and back-office duties influence the perception of some job characteristics and the environment and, consequently, their own well-being.

The Intra-class Correlation Coefficient (ICC) values of the variables analyzed in this study may be observed in Table 3, through which the proportion of variance explained by the organization for each duties, front-office and back-office, may be verified. As the number of participants in each organization differed, the Bonferroni test was conducted for each variance analyzed. As far as back-office employees are concerned, some of the job characteristics (job demands, autonomy, supervisor and peer support) of the work environment (recruitment, integration, training, performance assessment, compensation, and work–life conflict—stress dimension) and employees’ attitudes and stress and well-being (affective organizational commitment, work engagement, cynicism and general well-being—stress dimension), are observed to present significant differences among the various companies (ICC values ≥ 0.05). Thus, these dimensions are dependent on the company, suggesting that back-office work may vary according to the company in which the employee works. On the other hand, exhaustion, work–life conflict (time dimension) and general well-being (well-being dimension) appear to be common to all companies, since they do not present significant differences in variance (ICC values < 0.05).

As regards front-office duties, significant differences in variance in job characteristics (job demands, autonomy, peer support), work environment (recruitment, integration, training, performance assessment, compensation, and work–life conflict—stress and time dimension) and attitudes and stress and well-being (affective organizational commitment, cynicism and general well-being—stress dimension) may be observed. However, this is not the case for supervisor support, work engagement, exhaustion and general well-being, —well-being dimension), since no significant differences in variance are observed and, therefore they are common to all companies.

Thus, it may be concluded that feelings of exhaustion and general well-being in CC appear to be independent of the duties performed or of the companies in which employees develop their professional activity. On the other hand, the remaining job characteristics, namely those related to environment, attitudes and stress and well-being depend either on the duties performed or the company´s characteristics.

## 6. Discussion

This study sought to ascertain whether the characteristics of CC work are inevitable or whether they depend on the duties performed, namely front-office or back-office, and the company’s characteristics. It was possible to observe significant differences between the two functional groups: Front-office employees appear to have a more negative perception of autonomy and a greater perception of work–life conflict (stress dimension), consequently presenting worse levels of exhaustion. On the other hand, when comparing the results of fifteen different companies, job characteristics, environment, and levels of stress and well-being of the employees show significant differences, indicating that these characteristics are not inevitable in CC but rather depend on each company’s management strategy.

Regarding job characteristics, as expected, the CC context was found to be characterized by high demands and low control, resulting in high stress and low well-being levels [19,20,21]. However, front-office employees perceived less autonomy compared to back-office employees, in line with the assumption that the use of scripts that organize and structure the making of calls in the case of front-office duties [12] has negative repercussions for control in terms of the planning and organization of the tasks performed [8,11]. When comparing the professionals of these two groups, front-office employees presented higher levels of stress [67], namely exhaustion, which, in line with the role conflict theory [60], had an impact on a higher perception of work–family conflict (stress dimension).

On the other hand, this study managed to demonstrate innovatively that stressful characteristics [21] (high demands and low control) are not inevitable in the context of CC, as the data suggests variability among the companies and both back-office and front-office duties, showing that it is possible to reduce the workload and increase the autonomy of employees by redesigning these duties. In fact, the data of this study suggest that employees’ stress and well-being levels may differ [2,19] as the quantitative requirements that are associated with monitoring and performance assessment [12,54] and employees’ autonomy, giving them some freedom to plan and organize the tasks they perform [8,11], may also vary depending on their occupation and company.

Regarding social support on the part of the supervisor, this study identified a similarity among all the companies for employees with front-office duties. It suggests that the need to monitor and assess customer service may favor the standardization of supervisory duties among different companies. Given the knowledge that social support increases the resources required by employees to deal with high demand situations [47], this study highlights the need to promote the ability of supervisors to offer adequate social support in the context of CC [14].

On the other hand, the HR practices analyzed were considered to depend on the company. If these practices are considered fundamental to explain the results obtained in the context of CC [8], but also to explain the perception of the job characteristics themselves [15], then the following measures are sorely needed: Investment in appropriate recruitment and selection processes adapted to the duties [40]; investment in welcoming and integration programs that foster the creation of positive interpersonal relationships among employees [43]; provision of specific and planned training in order to increase employees’ resources [47]; adoption of a remuneration model that is compatible with the HIM model [49]; and the use of performance assessment as a diagnostic tool which aims to identify features requiring improvement [59].

As for work–life conflict, stress was considered to vary depending on the company and is, therefore, an avoidable variable. This suggests that a discrepancy between the demands of the domain in which the employee participates and the resources to which this professional has access [63] is not observed in all companies. Hence, and although employees have a relatively neutral perception of the work–life conflict, organizations should take measures to promote a balance between these two domains [60].

Regarding attitudes at work, affective organizational commitment was considered to vary depending on the company, and significant variance was observed in both front-office and back-office groups. Affective organizational commitment reflects the bond employees experience with their organization [66] and may be used as a tool to combat their stress [68]. As this positive attitude depends on the organizational context, namely the human resources management practices in place [32], and as the perception of these practices differs depending on the company in question, differences in attitude were also expected. However, and since the data suggests that participants have a neutral perception of affective commitment, it is imperative to focus on developing a positive emotional relationship between employees and their organizations [68] in order to promote their well-being [71].

As for well-being at work, differences in relation to burnout were observed between the two core dimensions of this chronic stress at work syndrome: Exhaustion appears to be cross-cutting and independent of the company and back-office or front-office duties; cynicism, conversely, appears to be dependent on the company for both back-office and front-office duties. This difference may be justified if the development of the burnout syndrome, as posited by the Conservation of Resources theory (COR, [97]), is taken into account. According to this theory, employees invest strongly in the acquisition of resources to meet the excessive demands with which they are confronted during their professional activity, resulting in a feeling of high exhaustion which characterizes a stress situation. Thus, working in a CC may be considered a highly demanding situation conducive to stress (i.e., exhaustion), as employees tend to perceive a loss of resources, the threat of resource loss or to invest in resources to face these demands. However, in order to cope with these same demands, employees use coping strategies, which may or may not trigger distancing responses, i.e., cynicism. If the context does not provide resources to protect employees from this sense of loss or threat of loss, they tend to drain their energy resources, and consequently, to protect themselves they will adopt an attitude of detachment which will result in cynicism, a characteristic of the burnout syndrome. However, if the context provides resources (e.g., control, social support, human resource practices that respond to employees’ needs), this stress situation may not become a burnout situation, as individuals do not need to adopt this cynical distance to deal with such situations of loss or threat of loss of resources.

With regard to work engagement, this positive psychological state appears to depend on the company for employees with back-office duties; however, it is cross-cutting and independent of the organization for front-office duties. Considering that work engagement is mainly dependent on the resources available to employees in the accomplishment of their tasks [76], in the case of back-office duties, there appear to be situations where the availability of resources varies, thus leading to variable degrees of work engagement, while in front-office duties the differences in resources are not sufficient to reflect differences in this indicator of well-being at work. In line with the results obtained, supervisor support was also found to be a resource that appeared not to differ among companies for employees with front-office duties. Since this resource has an extrinsic motivational role for being instrumental in the acquisition of work objectives, but also intrinsic for being able to satisfy the basic psychological need for relationships [98], it can play a central role in the development of these employees’ work engagement.

Finally, and analyzing general well-being, it is possible to observe that general stress is dependent on the company and for both back-office and front-office duties. This result shows that organizations can implement a number of strategies to mitigate employees’ stress. Furthermore, according to the burnout literature [99] and the health impairment process proposed by the Job Demand-Resource Model [76], burnout leads *to* different health problems outside the work context. As previously mentioned, although the levels of exhaustion (i.e., stress) are common to the different companies, their association with cynicism (i.e., burnout) is dependent on the company, hence the levels of stress outside the work context may also be dependent on the company.

In conclusion, this study appears to corroborate the idea that working in a CC implies experiencing stress in the accomplishment of one’s work, as the levels of exhaustion are independent of back-office and front-office duties and of the company in which one works. However, the development of burnout (i.e., cynicism) and general ill-being does not appear to be inevitable as the emergence of these conditions depends on the company in which the duties are performed. Moreover, job characteristics, peer support, HR practices, work–life conflict (in the stress dimension) and affective organizational commitment also appear to depend on the company. Thus, this study suggests that through a structured and planned intervention at the organizational level, it is possible to promote a healthier work environment that will foster the well-being of employees.

### Limitations and Future Implications

A number of limitations need to be addressed regarding the present study. First, the method used for data collection may have skewed the results, since a self-assessment questionnaire was used, which was disclosed internally by each company’s HR department. Thus, and despite the anonymity of the responses, they may contain some level of social desirability. Secondly, as this is a cross-sectional study, the data refers to a single point in time, which does not allow for the establishment of cause-effect relationships. Therefore, it was only possible to make inferences with regard to the assessment of positive or negative relationships between the studied variables. It would be interesting to conduct a longitudinal study in order to analyze any further developments in the perception of employees regarding the factors studied in this research, as well as to monitor intervention plans that may have been applied. Thirdly, the sample consisted only of employees from Portuguese CC, and it was not possible to generalize the results to other countries or other sectors of activity. In the future, it would be important to compare the results obtained in this study with those of other countries and/or other sectors that are dominated by customer service, such as the hotel industry or trade employees. Finally, the quantitative analysis used in this study does not allow for a comprehensive vision of employees’ experience in a contact center context. In the future, it would be interesting to conduct a qualitative study that could examine the meaning of the employees’ history in this context, analyzing their experiences (e.g., peer support climate, supervisor support strategies, learning of day-to-day problem-solving strategies) and respective repercussions for their well-being and health.

## 7. Conclusions

This study provides evidence that the specific nature of the duties performed by Contact Center employees, i.e., front-office and back-office duties, has an impact on how they perceive their job characteristics and environment and, consequently, on their well-being. In addition, it highlights that although the exhaustion and general well-being of CC are independent of their duties and common to all employees regardless of the company in which they work, job characteristics, psychosocial environment, and the levels of affective organizational commitment, cynicism, and general stress of CC employees depend on the company in which they work.

## Figures and Tables

**Table 1:** Sample Demographics’.

Sample	Total (*N* = 1440)	Front-Office (*N* = 787)	Back-Office (*N* = 653)
Age (mean)	32.31	31.30	33.53
Gender (% female)	63%	62.1%	64%
Level of schooling (%)			
Below Secondary	4.3%	3.7%	5.1%
Secondary	34.3%	34.9%	33.5%
Higher Education Attendance	22.8%	23.5%	21.9%
Degree	27.7%	28.1%	27.3%
Post-degree qualification	10.3%	9%	11.8%
Tenure			
Less than 3 months	8.3%	10.4%	5.7%
Between 3 and 6 months	12.2%	12.5%	11.9%
Between 6 and 9 months	9.2%	9.5%	8.9%
Between 9 months and 1 year	4.7%	4.2%	5.2%
Between 1 and 5 years	38%	37.5%	38.6%
Between 5 and 10 years	19.%	18.2%	21%
More than 10 years	8.2%	7.8%	8.7%
Number of working hours per week			
20 to 24 h	9.3%	11.8%	6.3%
25 to 29 h	4.5%	6%	2.8%
30 to 34 h	7.7%	9.4%	5.7%
35 to 40 h	78.5%	72.8%	85.3%
Marital Status			
Single	57.4%	59.3%	55.1%
Married or in a stable relationship	35.4%	35.7%	35.1%
Divorced or separated	6.8%	4.4%	9.8%
Widowed	0.3%	0.6%	0%

**Table 2:** Mean, Standard Deviation, and Student *t*-test of the Variables under Study, according to Occupation.

Variables	Back-Office (*N* = 653)		Front-Office (*N* = 787)		T Test	
	Mean	SD	Mean	SD	*t* *	*p*
**Job Characteristics**						
Job demands	3.5	0.82	3.55	0.81	−1.05	0.29
Autonomy	2.98	0.98	2.68	0.95	5.93	0.00
**Social Support**						
Supervisor Support	5.02	1.46	5.07	1.4	−0.72	0.46
Peer Support	5.02	1.24	5.05	1.15	−0.61 **	0.55
**HR Practices**						
Recruitment	5.13	1.37	5.17	1.25	−0.45 **	0.65
Integration	5.34	1.27	5.47	1.21	−1.98	0.48
Training	4.71	1.44	4.64	1.46	0.96	0.34
Compensation	3.6	1.64	3.97	1.59	−4.36	0.00
Performance Assessment	4.69	1.67	4.73	1.55	−0.54 **	0.59
**Work–Life Conflict**						
Time	3.24	1.03	3.33	1.01	−1.53	0.13
Stress	3.24	1.08	3.42	1.06	−3.14	0.00
**Attitudes and Well-Being at Work**						
Affective Commitment	4.26	1.56	4.34	1.47	−0.94	0.35
Engagement	4.39	1.63	4.28	1.57	1.30	0.19
Exhaustion	3.99	1.74	4.25	1.69	−2.79	0.01
Cynicism	3.09	1.64	3.19	1.65	−1.12	0.27
**General Well-being**						
Stress	2.9	0.76	2.81	0.76	2.14	0.03
Well-being	2.34	0.6	2.34	0.59	−0.08	0.94

* The *t* value was calculated by the mean difference between back-office and front-office employees. ** According to the Levene Test, equal variances were deemed not assumed.

**Table 3:** Intra-class correlation coefficient of the variables under study, by occupation.

Variables	Back-Office		Front-Office	
	ICC	*p*	ICC	*p*
**Job Characteristics**				
Work Overload	0.06	<0.001	0.11	<0.001
Autonomy	0.06	<0.001	0.08	<0.001
**Social Support**				
Management Support	0.09	<0.001	0.04	<0.001
Peer Support	0.05	<0.001	0.06	<0.001
**HR Practices**				
Recruitment	0.05	<0.001	0.06	<0.001
Integration	0.07	<0.001	0.08	<0.001
Training	0.06	<0.001	0.10	<0.001
Rewards	0.05	<0.001	0.09	<0.001
Performance Assessment	0.10	<0.001	0.05	<0.001
**Work–Life Conflict**				
Time	0.03	0.002	0.07	<0.001
Stress	0.06	<0.001	0.07	<0.001
**Attitudes and Well-Being at Work**				
Affective Commitment	0.10	<0.001	0.11	<0.001
Engagement	0.06	<0.001	0.03	0.001
Exhaustion	0.04	<0.001	0.04	<0.001
Cynicism	0.05	<0.001	0.08	<0.001
**General Well-being**				
Stress	0.05	<0.001	0.07	<0.001
Well-being	0.01	0.227	0.01	0.152

Note: *p* values are the result of the ANOVA performed to calculate the mean squares between and within groups; the Bonferroni test was used to correct the significance level.

## Data Availability

The data presented in this study are available on request from the corresponding author.
